# Fixation of fibular head avulsion fractures with the proximal tibiofibular screw: Technique guide and clinical experience

**DOI:** 10.1016/j.tcr.2025.101175

**Published:** 2025-04-28

**Authors:** Ryan A. Paul, Shu Yang Hu, Ananya Pathak, Ryan Khan, Daniel B. Whelan

**Affiliations:** a Department of Surgery, Temerty Faculty of Medicine, University of Toronto, Toronto, Ontario, Canada; b Division of Orthopedic Surgery, Department of Surgery, University Health Network (UHN), Toronto, Ontario, Canada; c University of Toronto Orthopaedic Sports Medicine Program, Toronto, Ontario, Canada; d Division of Orthopaedic Surgery, Department of Surgery, University of Toronto, 149 College Street, Room 508-A, Toronto, ON M5S 1A1, Canada; e Division of Orthopaedic Surgery, Women's College Hospital, Toronto, Ontario, Canada; f Division of Orthopaedic Surgery, St. Michael’s Hospital, Toronto, Ontario, Canada

**Keywords:** Posterolateral corner injury, Fibular avulsion fractures, Knee, Multiligamentous knee injury

## Abstract

**Background:**

Fibular head avulsion fractures are often associated with posterolateral corner injuries. Conventional fixation have consisted of transosseous or anchor-based suture techniques.

**Purpose:**

This technique paper reports on the use of a cannulated screw for fixation of fibular head avulsion fractures by a single surgeon at a tertiary referral center.

**Methods:**

Thirty-seven patients underwent open reduction and internal fixation of fibular head avulsion fractures between 2006 and 2022.

**Results:**

At final follow up (mean 3.5 years ± 2.5) all fractures went on to bony union. All patients regained functional range of motion with mean extension of 1 degree (median 0 degrees, range 0 to −5 degrees) and mean flexion of 121 degrees (median 127.5 degrees, range 90 to 135 degrees).

**Conclusion:**

Our results suggest that a cannulated screw across multiple cortices provides robust fixation and allows for early motion.

## Introduction

Fibular head avulsion fractures are uncommon but significant findings often associated with multiligament knee injury [[1], [2], [3]]. The incidence is approximately 0.6 % after an episode of knee trauma [4]. The fibular head is the insertion site of multiple components of the posterolateral corner including the lateral collateral ligament (LCL), biceps tendon (BT), popliteofibular ligament and arcuate ligament [5]. As such, avulsion injuries of the fibular head have been termed “arcuate fractures” [4,6].

When present, arcuate fractures are a sign of posterolateral corner instability. Varus laxity is present primarily due to disruption of the LCL, while incompetence of the remaining structures result in increased external rotation and posterior translation of the tibia. Additionally, up to 72 % of arcuate fractures are associated with cruciate ligament injury [7].

Surgical fixation of acute fibular head avulsion fractures is indicated to restore stability, particularly when addressing concomitant injuries [8]. Failure to do so may result in ongoing instability, increased stress on repaired or reconstructed cruciate ligaments, and may increase the likelihood of chronic degenerative changes. Previously published techniques for fibular head fixation have consisted of transosseous or anchor-based suture techniques [[8], [9], [10], [11], [12], [13], [14]]. However, we prefer utilizing a cannulated screw with fixation across multiple cortices as we have found that this provides more robust fixation and allows for early motion. Biomechanically, screw fixation has been shown to be superior to transosseous suture repair in arcuate fractures [15].

The purpose of this article is to report on the use of a cannulated screw for fixation of fibular head avulsion fractures by a single surgeon at a tertiary referral center between 2006 and 2022. Our hypothesis is that this technique provides secure fixation allowing early range of motion, restored of stability of the posterolateral ligament complex, and will be associated with high rates of union and a low incidence of complications.

## Methodology

### Study design

This study employed a retrospective case series to evaluate the outcomes of fibular screw fixation in 37 patients undergoing open reduction and internal fixation (ORIF) from 2006 to 2022, often concurrently with cruciate ligament reconstruction, meniscal repair, and/or tibial plateau fixation.

### Participants

Thirty seven patients were included in the study. Inclusion criteria consisted of patients who exhibited varus stress test laxity grade 2 or greater, along with imaging confirmation of a displaced fibular fracture and associated retraction of the arcuate ligament complex. Patients requiring concomitant reconstruction of the lateral collateral ligament (LCL) or posterior cruciate ligament (PCL) were excluded. All patients underwent preoperative radiographs, MRI, and, in certain cases, CT imaging for detailed assessment of associated fractures, particularly tibial plateau fractures.

### Interventions

All participants underwent open reduction and internal fixation of the fibular fracture. Surgical procedures also included concurrent cruciate ligament reconstruction, meniscal repair, and/or tibial plateau fixation as deemed necessary during the same operation.

### Postoperative management

Following surgery, all patients were immobilized with splints and were instructed to engage in touch weight bearing for a period of 6 weeks. Early range of motion (ROM) exercises were initiated at 2 weeks postoperatively. The final follow-up evaluation was conducted at an average of 3.5 years post-surgery, during which clinical and radiographic examinations were performed to assess outcomes.

### Outcome measures

The primary outcomes assessed included clinical measures such as stability as determined by varus stress testing and radiographic evaluation of fracture healing. Secondary outcomes included functional recovery assessed through patient-reported outcomes and return to daily activities.

### Data analysis

Descriptive statistics were used to summarize demographic data and clinical characteristics of the patients. Continuous variables were reported as means with standard deviations or medians with interquartile ranges, as appropriate. Categorical variables were summarized as frequencies and percentages.

### Surgical technique

All operations were performed under combined regional block and general anesthetic. Patients were placed supine with a pneumatic tourniquet applied to the ipsilateral thigh. A lateral bolster and sandbag were used to allow positioning in 90 degrees of flexion. Examination under anesthesia confirmed the presence of varus and rotational laxity as well as associated injuries. Following completion of tibial plateau fracture fixation and/or preparation of cruciate ligament reconstruction, the fibular fracture was approached.

**Fig. 1:**
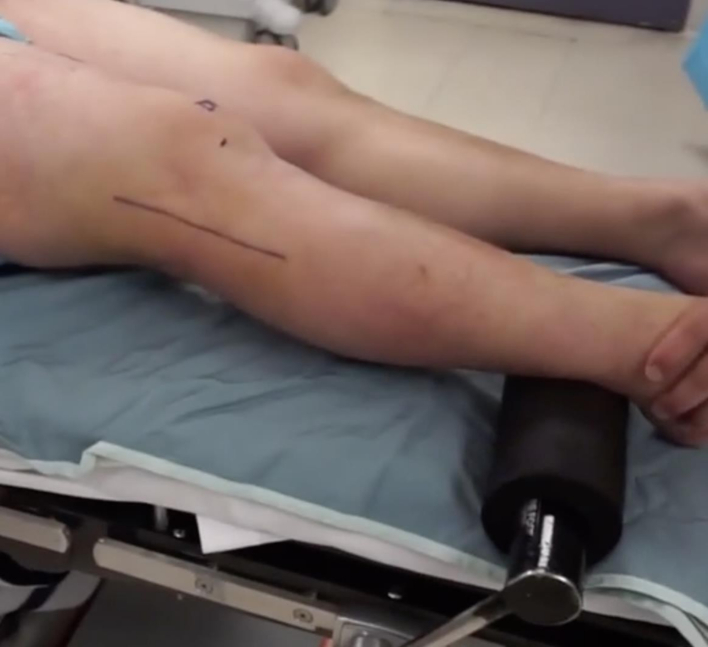
Lateral based incision in extension.

**Fig. 2:**
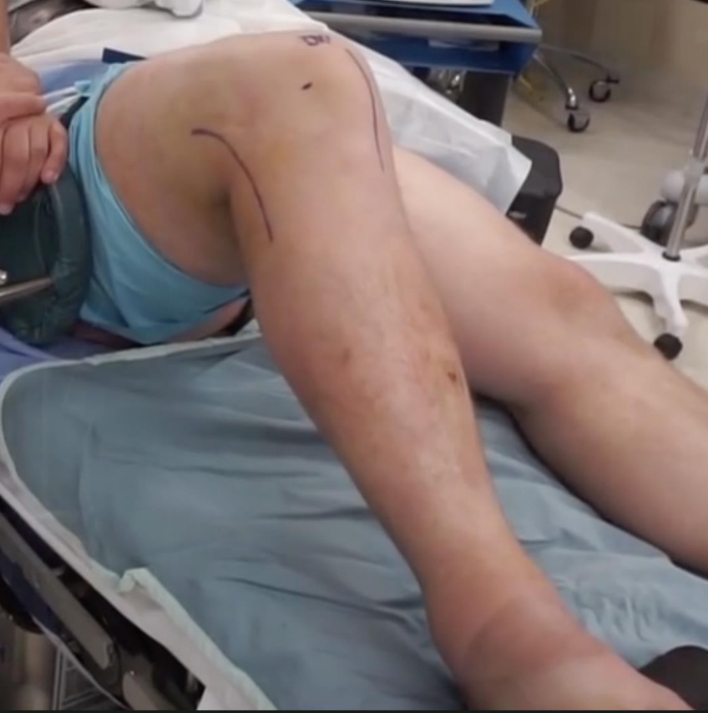
Lateral based incision in flexion.

**Fig. 3:**
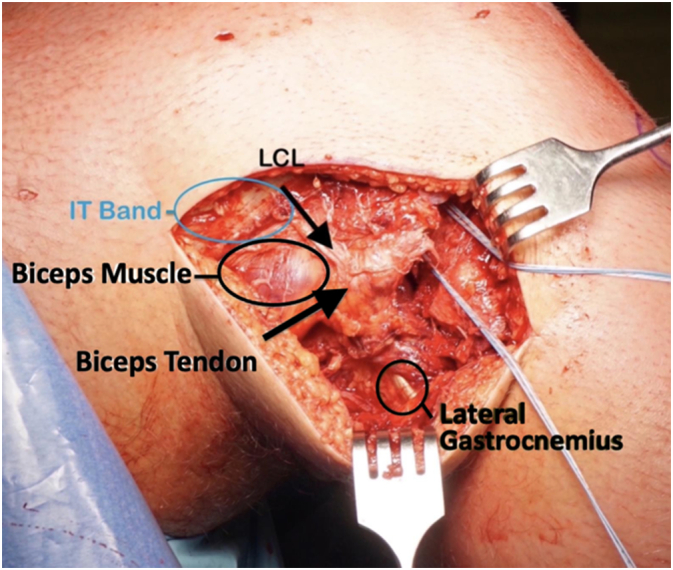
Dissection carried down to IT band. Blunt dissection of soft tissue structures.

A lateral based incision was made extending from the lateral epicondyle to a point approximately 2 cm distal to the fibula along its anterior border. The incision was designed to be longitudinal with the knee in full extension (Fig. 1) and curvilinear with the knee flexed (Fig. 2). Dissection was carried down sharply to the level of the iliotibial band. Blunt dissection was performed posteriorly where the fibular head fracture and attached ligamentous structures were identified (Fig. 3). The common peroneal nerve was found posterior to the biceps at the level of the fibular neck and exposed proximally and distally along its length, past the fracture site and area of fibular screw insertion. This was done to confirm that the nerve was intact since injury, and also to prevent iatrogenic damage. The nerve was isolated and protected throughout the case with the use of a Penrose drain. A small window was then made proximally in the IT band in order to confirm intact attachments of the LCL and popliteus on the lateral condyle. When necessary, attachments were reinforced with suture anchor repair.

**Fig. 4:**
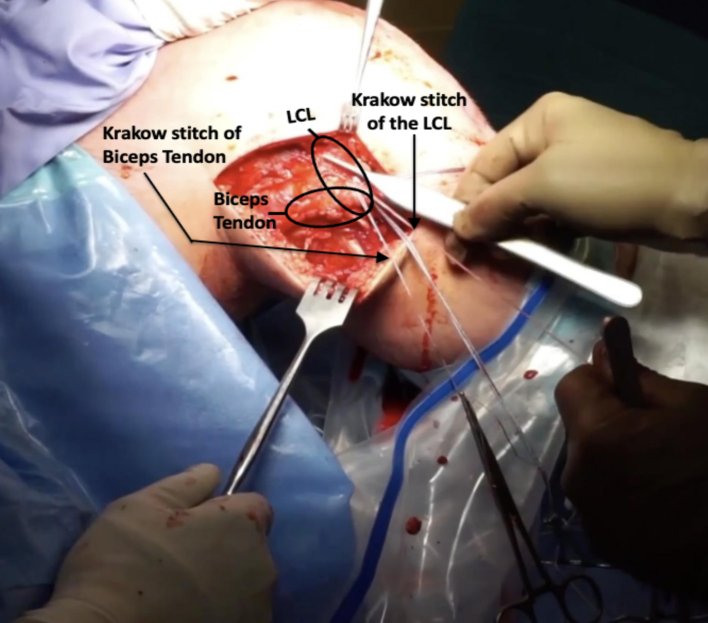
A running locking stitch (Krakow) of the LCL and biceps tendon before fixation.

**Fig. 5:**
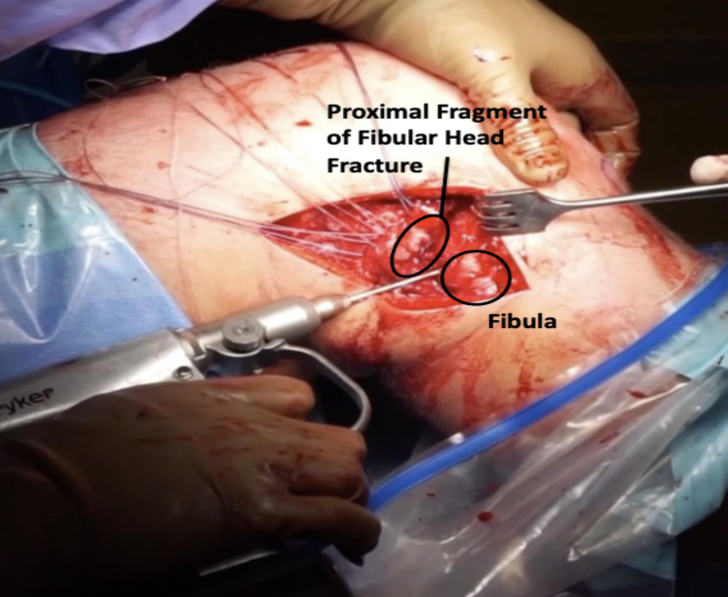
Guide wire placed obliquely from the posterior lateral aspect of the fibula, distally and anteriorly through the tibia.

Fibular fixation was then performed. A running locking Krackow stitch was performed using high strength suture in the lateral collateral ligament as well as the biceps tendon providing secure fixation (Fig. 4). Blunt dissection was then performed to mobilize the soft tissues and allow reduction of the fracture fragment. After this, a guidewire was placed obliquely from the posterolateral aspect of the fibula directed in an oblique orientation distally and anteriorly through the tibia (Fig. 5). When the fibular fracture was of sufficient size, the fracture was reduced and the wire was passed through the piece. For smaller fragments, the wire was placed first and the fragment was then captured below the screw head and washer.

**Fig. 6:**
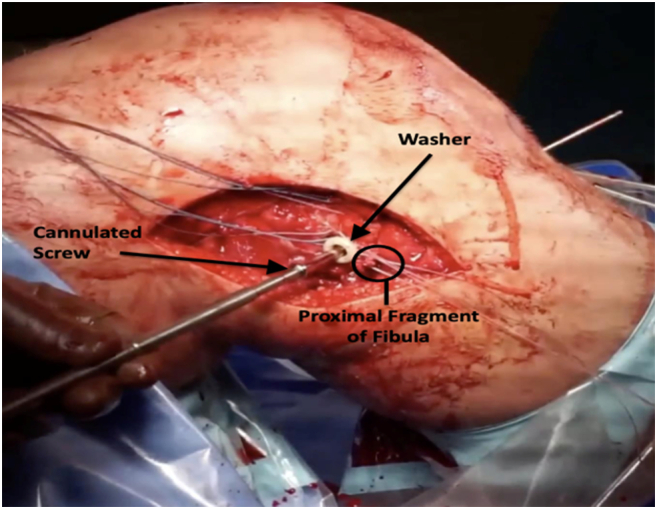
Spiked washer added with cannulated screw to facilitate capture of the proximal fibular fragment and soft tissue attachments. Krakow sutures were tied around screw providing additional fixation.

**Fig. 7:**
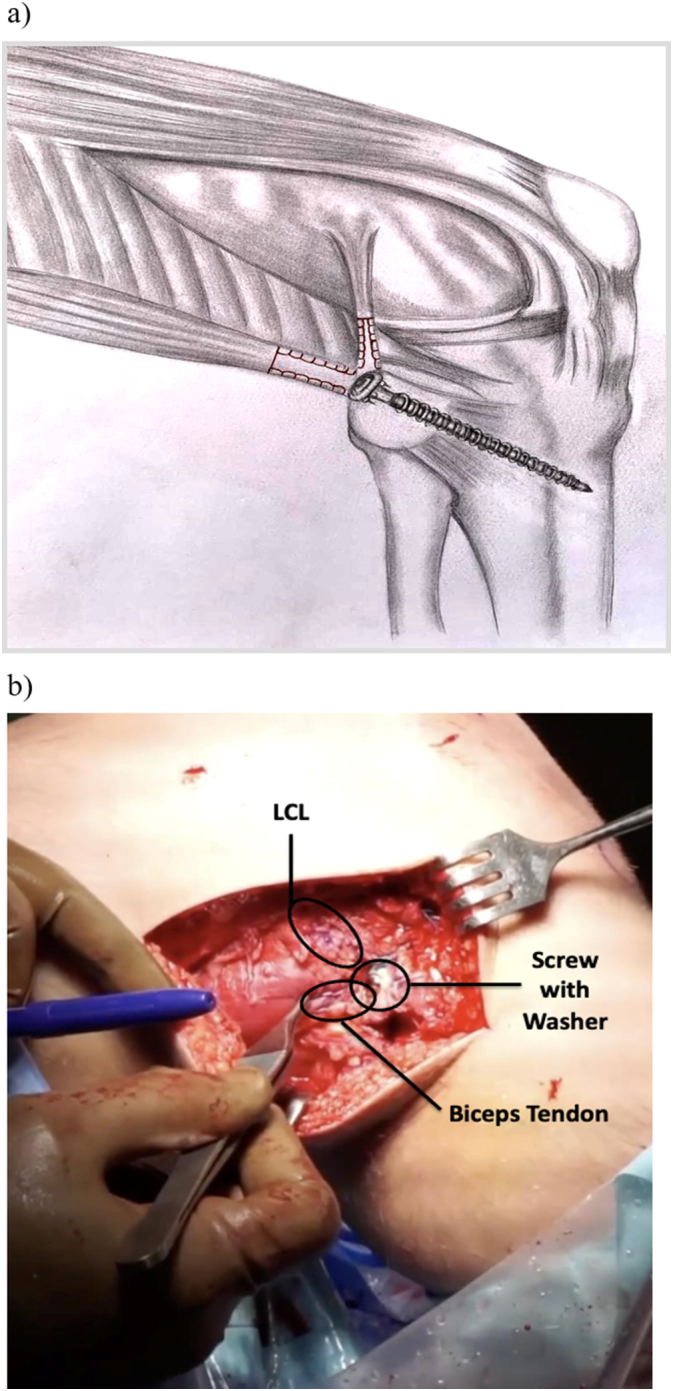
a) Schematic diagram of the proximal tibiofibular screw fixation. Krakow sutures (shown in red) are placed in the lateral collateral ligament (LCL) and biceps tendon, then secured around a screw/soft tissue washer passed through the fibula into the tibia. b) Final intraoperative image showing proximal fibular fragment screw fixation with washer with stabilization of LCL and biceps tendon. (For interpretation of the references to colour in this figure legend, the reader is referred to the web version of this article.)

Once the wire was in place, it was over-drilled, and a 6.5 or 7.0 mm cannulated large fragment screw was inserted. A spiked soft tissue washer was added to facilitate capture of the fibular fragment and soft tissue attachments (Fig. 6). Prior to final tightening, the Krackow sutures were tied around the screw providing additional fixation (Fig. 7).

After fibular fixation, cruciate ligament reconstructions underwent final tensioning and fixation. Additionally, lateral meniscocapsular detachment was assessed and repaired with suture anchor fixation as needed. All wounds were then copiously irrigated and closed in layers. Sterile dressing was applied and a postoperative splint was fitted with slight valgus mold.

## Results

Thirty-seven patients underwent open reduction and internal fixation of fibular head avulsion fractures between 2006 and 2022 using a large fragment cannulated screw and soft tissue washer inserted obliquely from the proximal fibula to tibia. All patients had a multi ligamentous injury. Mean age was 36.8 ± 13.6 years. There were 32 male and 5 females. Mechanism of injury included 18 motor vehicle accidents, 4 falls, and 13 sports or fighting related injuries.

**Fig. 8:**
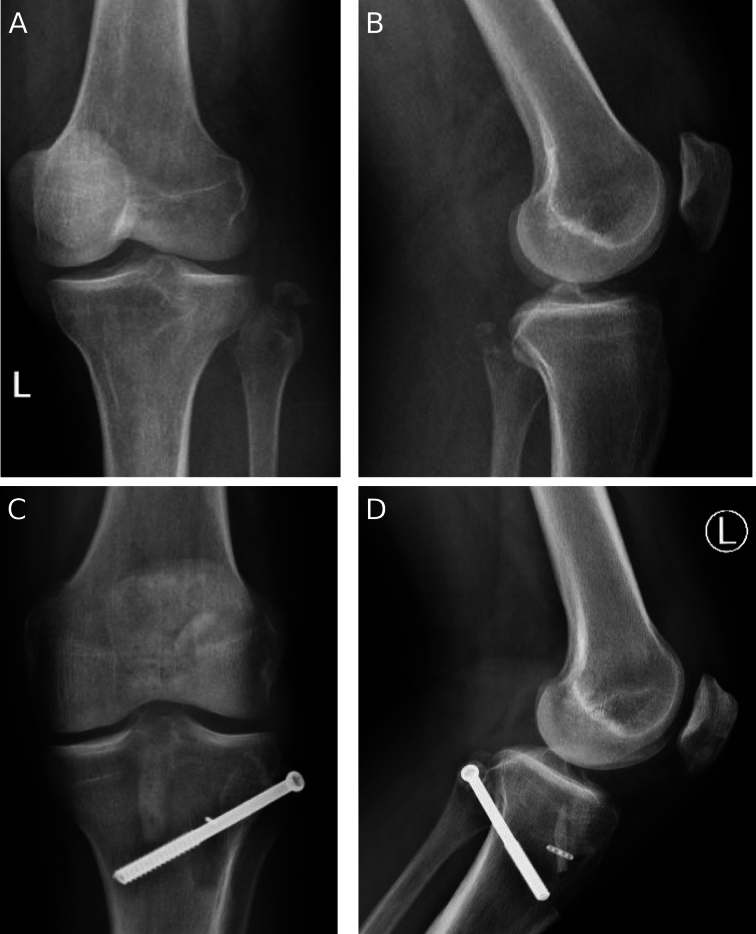
Pre-operative anteroposterior (a) and lateral radiographs (b) of a fibular avulsion fracture. Post-operative anteroposterior (c) and lateral radiographs (d) demonstrating the proximal tibiofibular screw fixation.

All 37 patients returned for clinical and radiographic assessment. At final follow up (mean 3.5 years ± 2.5) all fractures went on to bony union.(Fig. 8) No patients reported pain or instability at the proximal tibiofibular joint. There were no reoperations for recurrent instability, and no patient required lateral or posterolateral ligament reconstruction. All patients regained functional range of motion with mean extension of 1 degree (median 0 degrees, range 0 to −5 degrees) and mean flexion of 121 degrees (median 127.5 degrees, range 90 to 135 degrees). Two patients described asymptomatic hardware prominence that did not require intervention. One patient had a symptomatic palpable screw removed. One patient had screw back out of approximately 5 mm on radiographs and subsequently had this removed after fracture union. One patient developed a late local infection to the lateral incision, which occurred greater than 5 years after surgery and required debridement. Four patients developed asymptomatic heterotopic ossification around the fibular fracture.

Fracture morphology was assessed based on preoperative radiographs and cross-sectional imaging. In our patients, 90 % of fractures demonstrated an oblique pattern in the coronal plane (with the fracture oriented from proximal-medial to distal-lateral). In the sagittal plane, the fracture pattern was transverse in 80 %.

## Discussion

Fibular head avulsion fractures are a sign of significant ligamentous knee instability and are highly associated with concomitant injuries [[1], [2], [3],^16^]. Acute fractures should be treated with stable fixation, allowing early range of motion to optimize patient outcome. Immobilization after tenuous repair may be associated with capsular contracture and post-traumatic stiffness. Previously published fixation techniques have typically consisted of transosseous or anchor-based suture repair [8,[10], [11], [12], [13], [14]]. Strengths of the proposed technique include the use of a standardized cannulated screw. All patients went on to fracture union and achieved good postoperative range of motion, particularly with regaining terminal extension.

Geeslin and Laprade reported on a series of 29 patients with acute grade III posterolateral knee injuries [8]. Unlike the current study, their approach primarily involved reconstruction or a combination of reconstruction and repair; only two patients had fibular collateral ligament repair without reconstruction utilizing a suture anchor technique. Nevertheless, they describe good results with significant improvement in International Knee Documentation Committee (IKDC) scores [17] (from 29.1 to 81.5) and restoration of varus stability on stress radiographs compared to the contralateral side at an average 2.4 year postoperative followup. Oh et al. reported on a small series of seven consecutive patients treated with bioabsorbable suture anchor repair in addition to management of concomitant ligament injuries [11]. They reported 100 % fracture union, no complications, and no recurrent instability at the two year post-operative mark. Mean postoperative IKDC score was 89.6 points (range, 85–93).

Our preference is to use a large fragment cannulated screw across multiple cortices in order to provide more robust fixation of these difficult injuries. The addition of a soft tissue washer and suture repair around the screw allows for improved stability, particularly in very small or comminuted fragments. Biomechanically, we believe that screw fixation is superior to suture-based repair techniques. Vojdani et al. demonstrated that arcuate fracture fixation with an intramedullary screw and spiked washer results in at least two times the stiffness, failure force and energy to failure compared to transosseous suture repair in cadaveric testing [15]. While their technique differs from ours, we believe that the addition of cortical fixation of both the fibula and tibia will result in increased construct strength and very reliable initial fixation.

We do note that there was a 20 % (4/20) rate of hardware-related issues in our patients. This involved asymptomatic prominence in two patients (not requiring treatment), and two patients who underwent screw removal. This may be attributed in part to the use of a large fragment screw and, as such, one may consider smaller caliber screws as an alternative – however we do not have experience with this specifically.

Large fragment screws were chosen for a number of reasons. Firstly, there is a significant amount of tension placed on the fixation construct, particularly in delayed/subacute cases where there is reduced soft tissue excursion and given the strong dynamic pull of the musculotendinous structures. Secondly, the screw has to cross four cortices (which can be relatively thick on the tibial side) and this may cause deformation or deflection of a smaller caliber screw and predispose to breakage. Lastly, a larger screw allows for the placement of a more robust washer in order to secure otherwise small and/or friable fracture fragments and is better able to capture the critical soft tissue component of these injuries.

Radiographically, arcuate fractures have typically been identified as a transverse fracture of the fibular head with proximal retraction [8]. In our study, however, close examination of radiographs and cross-sectional imaging demonstrates an oblique morphology in the coronal plane (from proximal-medial to distal-lateral), while typically demonstrating a transverse morphology in the sagittal plane. This coronal fracture orientation makes it ideal for screw fixation as per the described technique.

## Conclusion

We have used this fixation technique of a large fragment cannulated screw for fibular head avulsion fractures routinely in our practice. Specifically, it is placed obliquely from the fibula to tibia and augmented with a soft tissue washer and suture repair. Our results suggest that this technique allows for high rates of union and early range of motion with maintenance of reduction.

## CRediT authorship contribution statement

**Ryan A. Paul:** Conceptualization, Data curation, Formal analysis, Methodology, Writing – original draft, Writing – review & editing. **Shu Yang Hu:** Data curation, Formal analysis, Methodology, Visualization, Writing – review & editing, Writing – original draft. **Ananya Pathak:** Data curation, Resources, Visualization, Writing – review & editing. **Ryan Khan:** Data curation, Resources, Writing – review & editing. **Daniel B. Whelan:** Conceptualization, Data curation, Resources, Supervision, Writing – original draft, Writing – review & editing.

## Declaration of competing interest

None.
